# Singularity index and multifractal analysis of magnitude magnetic transforms: a new methodology to explore Au mineralization with application to Esh El Mallaha, Egypt

**DOI:** 10.1038/s41598-025-93461-z

**Published:** 2025-03-31

**Authors:** Mohamed Nazih, Mohamed Mostafa Gobashy, Hossam Khamis, Mohamed A. El-Sadek, Khaled Soliman Soliman

**Affiliations:** 1 Faculty of Science, Geophysics Department, Cairo University, Giza, Egypt; 2 Nuclear Materials Authority, Maadi-Kattamia Road, P.O. Box 530, Cairo, Egypt

**Keywords:** Magnetic exploration, Mineralization, Magnitude magnetic transforms (MMTs), Fractal/multifractal analysis, Singularity index of MMTS, Solid Earth sciences, Geomagnetism

## Abstract

The primary objective of mineral exploration is to discover new mineral-rich zones within targeted regions. The fractal concentration area (C-A) and the magnetic maps are now extensively used in mineral prospecting. Unfortunately, the calculation of the reduced-to-pole (RTP) maps suffers from several drawbacks. It requires a prior knowledge of the inclination and declination of the source magnetization. It can be complicated to determine the direction of the source magnetization vector in certain conditions because of the large and significant remanent magnetization of the source if present. Furthermore, at low magnetic latitudes, the RTP computation is unstable. However, a new class of transforms known as magnitude magnetic transforms (MMTs) overcome these drawbacks. Such transforms have nonnegative distributions and exhibit significantly higher centricity with regard to the observed anomalous field. Their anomaly patterns are much less influenced by the direction of the magnetization vector than the observed total magnetic intensity. Due to these benefits, in this work, these transforms are used instead of RTP transform as a base for fractal/multifractal analysis of the magnetic signal from the Esh El Mallaha area, Eastern desert to delineate gold mineralization and hydrothermally altered and potential zones. Moreover, are used for the singularity analysis S-A in a novel integrated workflow. The results of this study show a promising approach that can be utilized globally for mineralization detection strategies.

## Introduction

Subsurface magnetic sources are magnetized by the Earth’s inducing field, which produces induced magnetization coordinated with the geomagnetic field. Additionally, another component of magnetization is called natural remnant magnetization (NRM), which is the permanent magnetism of a rock or sediment, may be present. This NRM usually differs from induced magnetization and can be complicated to determine. The total magnetization of the rock is simply the vector sum of both components. Hence, it is of paramount importance for accurate interpretations and inversion of magnetic data to recognize the type of magnetization in advance or use filters independent of the Declination (D) and Inclination (I) of source bodies.

On the other hand, mapping the boundaries of magnetized objects can be achieved when employing high-resolution magnetic data. Generally, delineating the lateral magnetization can offer analysis of lithological changes, structural regimes, tectonic styles, and trends, making it a useful guide for field mapping and alteration zones identification not only spatially but also in the third dimension, the depth^1^. Additionally, magnetic interpretation frequently makes use of contact or structure-mapping techniques that use many gradient-dependent filters, e.g. the analytic signal AS^2^, the local wave number LWN^3^, and the horizontal gradient magnitude of the tilt HG^4^. All of these functions depend on high-order derivative estimates and are consequently highly dependent on the accuracy of the data. The RTP transform is the basic magnetic transform used for most of these filters. It is used instead of total magnetic intensity because it removes the skewness due to the geomagnetic field polarities. However, as Gerovska and Bravo^5^ stated, the primary restrictive assumption when using the RTP transforms is that only induced source magnetization is considered, suggesting that (I_0_ = I), and (D_0_ = D), where I_0_ and D_0_ are those of the ambient geomagnetic field. Because of this presumption, RTP field maps will be inaccurate and eventually will lead to inaccurate qualitative and quantitative interpretations. This is particularly problematic since instead of only changing the observed magnetic field, many inversion techniques, such as those employed by Williams et al.^6^, used the RTP field.

In the past few decades, airborne geophysical methods (especially aeromagnetic and aero radiometry) have become more and more common in the field of mineral prospecting, specially at regional surveys where there are few rock outcrops and complex geological variables that affect the region and introduce errors into the data. These airborne techniques can reduce the time and enormous expenses associated with exploration by covering a vast region quickly and accurately^7–12^.

Fractal and multifractal approaches have recently been applied to these large-scale airborne geophysical data. They provided conclusive and promising results^13,14^. Additionally, through the integration of multifractal inverse distance weighting (MIDW), the concentration-area (C-A), and singularity analysis (S-A) techniques with airborne magnetometric data for discovering geophysical anomalies, these sophisticated statistical methods have succeeded in the identification of possible mineralization zones^13,15^. RTP, which was first suggested by^15^, is by default used in these integral studies (e.g.: as in^5^). However, due to the limiting assumption of the transformed RTP map, there may be errors in the resulting interpretation.

Consequently, the present work aims to present more suitable base magnetic transform maps that can be used for further processing to enhance the accuracy of mineralization exploration. These filtered maps are less dependent on the source body magnetization. The present study considers the Fractal/multifractal analysis and singularity analysis applied to the magnitude magnetic transforms (MMTs) as base transform magnetic maps, a suitable choice for this target. The total magnetic anomaly (TMA), sometimes referred to as the total magnetic intensity (TMI) is used to estimate the (MMTs)^16^. The Laplacian of the TMA (L), the modulus of the total gradient of the TMA (R), the square root of the product of the TMA and its Laplacian (Q), and the half square root of the Laplacian of the square of the TMA (E) are the components of the MMTs. These transformations show greater centricity regarding related sources and possess non-negative distributions compared to the observed anomalous field. Additionally, the orientation of the magnetization vector has a notably smaller effect on the anomalous patterns created by these transforms compared to the TMA itself^16–19^. A multifractal analysis will be utilized to assess these transformations to identify the most beneficial transform for gold exploration.

**Fig. 1:**
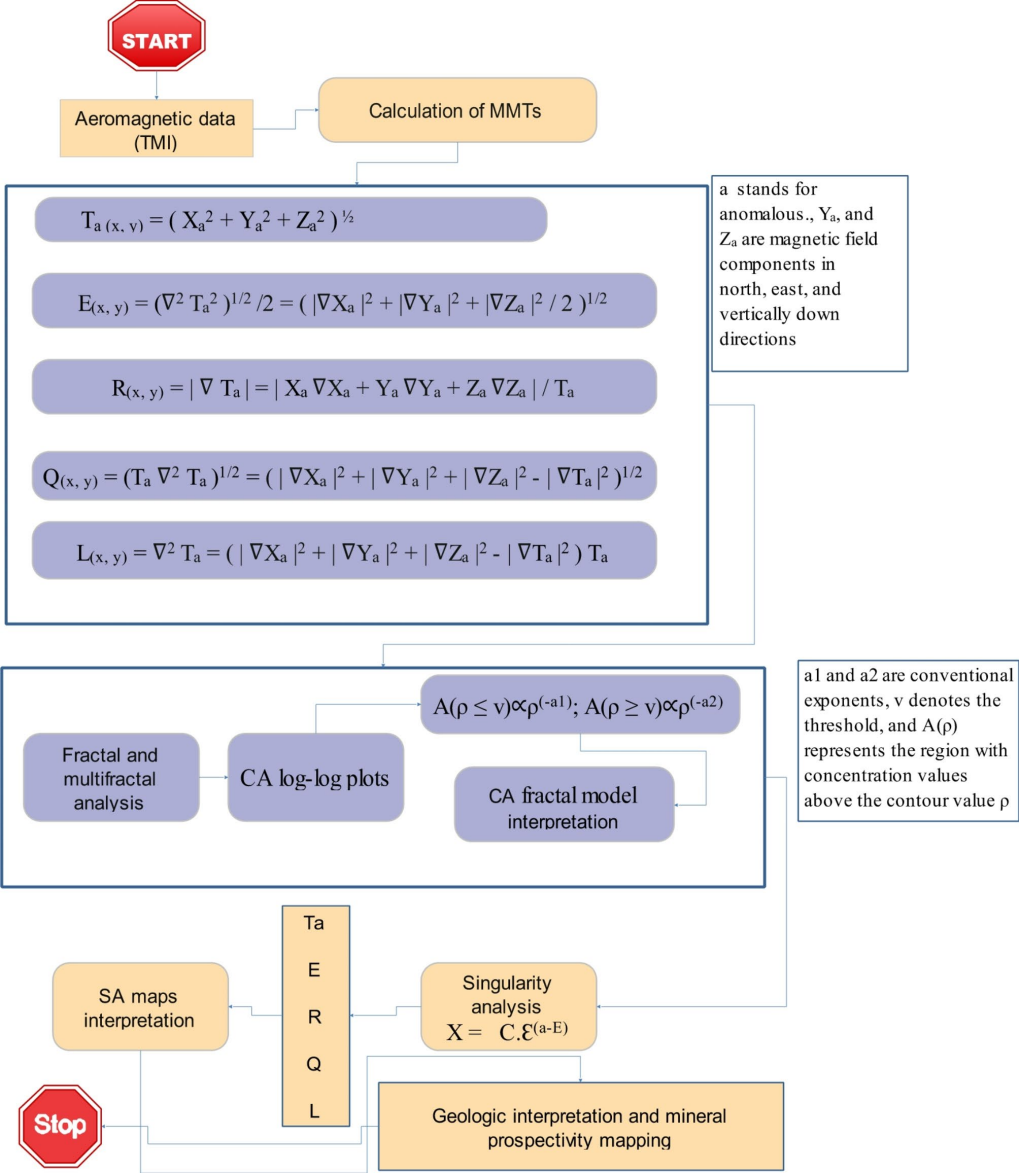
Proposed workflow utilizing MMTs, multifractal analysis, and singularity index for mineral exploration.

The workflow (Fig. 1) utilizing singularity index and multifractal analysis of Magnitude Magnetic Transforms is a novel methodology to explore mineralization deposits applied to Esh EL Mallaha regions (EM). The aeromagnetic survey^20–22^ conducted in the EM area is used to demonstrate the validity and applicability of this proposed workflow.

## The area of study

**Fig. 2:**
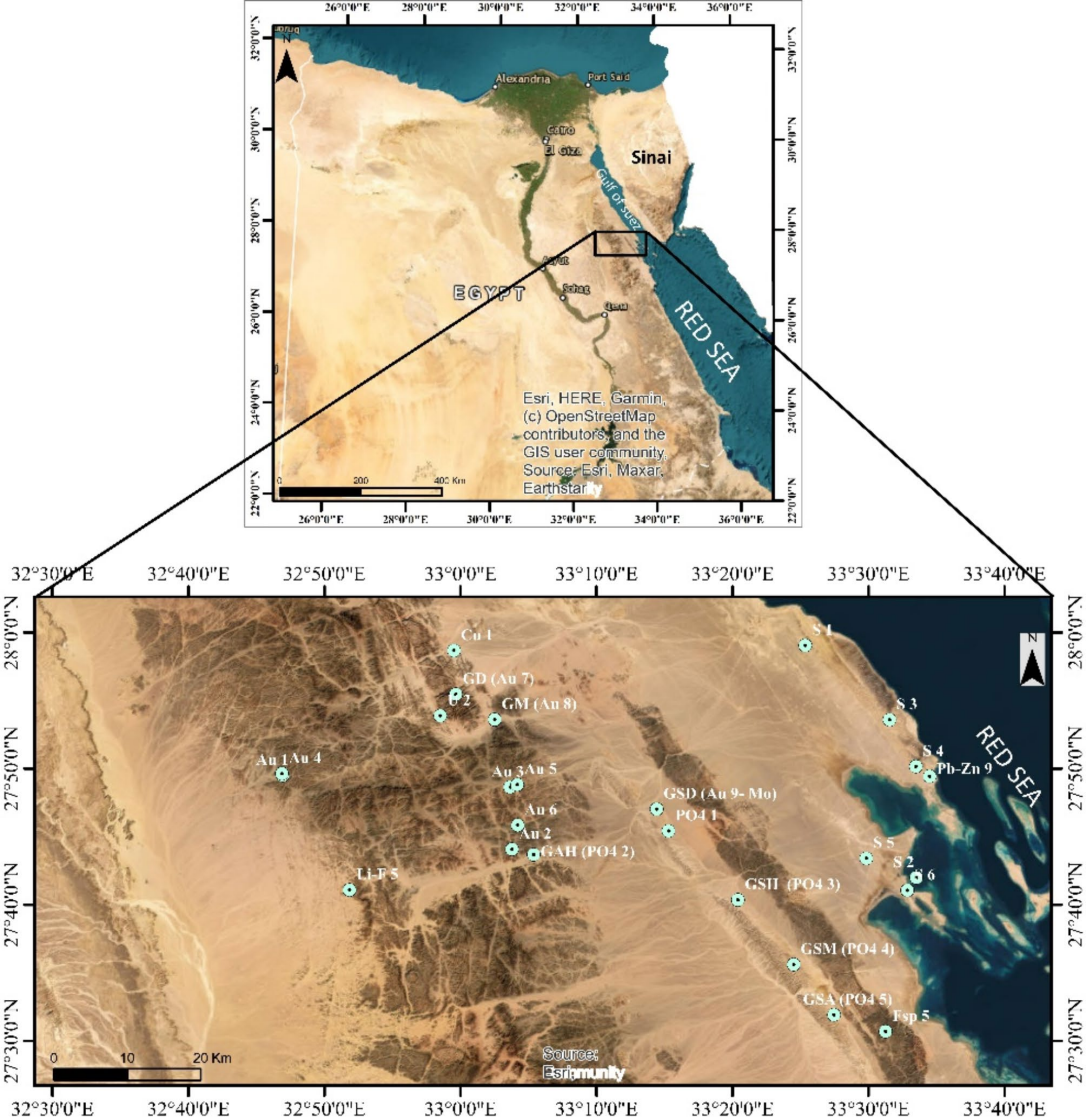
Location map of the Esh El Mallaha area. GD: Gabal Dara, GM: Gabal Monqul, GAH: Gabal Abu Had, GSD: Gabal Sufr El-Dib, GSH: Gabal Sufr Abu-Had, GSM: Gabal Sufr El-Milaha, GSA: Gabal Sufr El- Ash, S: Sulpher, PO_4_: Phosphate, Au: Gold, Fsp: Feldspare, Cu: Copper, and Pb-Zn: Lead-Zinc. To create the maps, ArcGIS Desktop version 10.8 was used. https://www.esri.com/en-us/arcgis/geospatial-platform/overview.

The Esh El-Mallaha (EM) is situated between latitudes 27° 30′ 00′′ and 28° 00′ 00′′ N and longitudes 32° 30′ 00′′ and 33° 40′ 00′′ E, in the northern portion of Egypt’s Eastern Desert. As illustrated in (Fig. 2), its surface area is approximately 7,084.5 km^2^. Graphite, Au, Cu-Ni, U, Mo, Fe, and phosphate are among the historical large-scale mineral accumulations in this region that are associated to hydrothermal processes^23,24^.

**Fig. 3:**
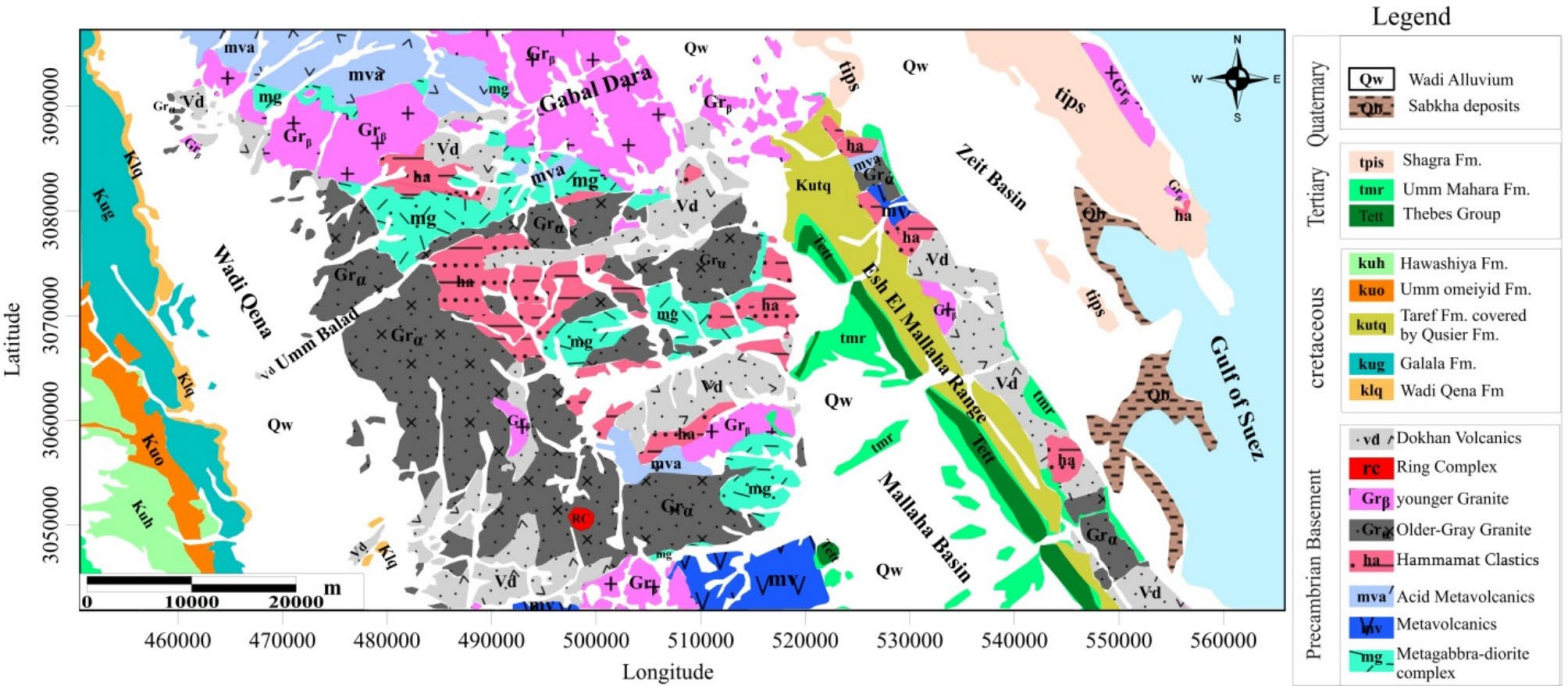
Geological map of EM area, NED, Egypt^32^.

The Eastern Desert (ED) of Egypt is separated into three distinct regions depending on basement characteristics^25^. The EM region is in the northern section of the ED (NED). The Gebal El- Zeit (GZ) and the Red Sea Hills, two elevated geological features, surround the region to the east (Figs. 2 and 3). Sedimentary and basement rocks are present on the surface in this location. This region is structurally divided into two major sedimentary basins, the Zeit and Mallaha basins, by the Esh El Mallaha (EM) range, a high topographic feature. The basement depths of the two basins, which have a NW-SE trend, range from 1.5 to 4.5 km. Gebal El-Zeit (GZ) borders the Zeit basin from the east, while the (EM) range borders it from the west^26^. Both basins extend from northwest to southeast and have basement depths of around 1.5 to 4.5 km^27^. Additionally, the Red Sea hills (RSH) and the (EM) range on its east and west flanks surround the Mallaha basin. A sequence of normal faults that run NW-SE surrounding the (GZ) and (EM) ranges^28^. These characteristic faults led to the development of the rocks of the (EM) range as well as younger sedimentary rocks. The rocks of the (EM) sector range in age from Precambrian to Recent, and they are a part of the Nubian shield^29^.

### Geological settings

Several geological units may be identified in the EM region, such as the metagabbro-diorite complex, granites, Dokhan volcanics, and Hammamat sediments, as illustrated in (Fig. 3). In obvious intrusive contacts, the younger granites regularly intruded into the older granites and gabbroic rocks; these plutonic rocks existed together with intramountainous Hammamat molasses sediments as the Dokhan volcanics extruded^30^. Monzogranites, which are found in minor outcrops in the northern part of the EM region, were the major kind of recently formed granitic rocks. Tonalite to granodiorite intrusions that have intruded into gabbroic rocks, creating intrusive sharp contacts, represent the older granites. The Dokhan volcanics are depicted by phenocrysts of plagioclase, quartz, and K-feldspar that are hidden inside a fine to medium-grained groundmass of biotite, quartz, and K-feldspar^31^.

The metagabbro–diorite complexes were essentially scattered throughout the EM zones. With minor quantities of quartz and alkali feldspars, the majority of these granular, coarse-grained textures are represented by plagioclase, biotite, and hornblende. They have alternating and rhythmic lamination between mafic to felsic layers, as well as small pyroxenite masses. Pyroxenite and amphibolite are found in many zones in these rocks. The metagabbro rocks in the NNE–SSW and N–S trends typically have an abundance of cracks and structural patterns disrupted^33^. Several veins and veinlets of smoky and milky quartz cross-cut the fundamental dykes, which are mostly covered with iron oxides and malachite. The excavated cracks in the shearing zones of the Cu-rich dykes in the metagabbro are expected to reveal an Au-Cu relationship inside the heterogeneous metagabbro-diorite.

The structure of the dykes that cut through the basement complex of the EM area and its surroundings were identified as Phanerozoic dykes, and there was an obvious relationship between their orientation and the main fault sets. These dykes are divided into two groups: bostonite dykes that trend in the NW-SE, NNW-SSE, and N-S, and basalt, andesite, and rhyolite dykes that primarily tendency in the NE-SW, ENE-WSW, and E-W trends. It is therefore concluded that these dykes were formed by simple differentiation from two magma sources^34^. It has been proposed that the anataxis of pre-existing crustal material during the late-collision stage peraluminously invaded the granites in the western region of EM^34,35^.

According to^36^, the primary deformation phase that produced the radioactive uranium anomalies in the pegmatite of the younger granites in the EM area was the NW-SE extensional event. Younger granite, Dokhan volcanic, Hammamat sediments, older granites, metavolcanic, acid-metavolcanic, and the predominant metagabbro-diorite rocks cover the western portion of EM, which includes multiple mining zones. Different dykes, mineralized veins, and quartz veins distinguish these rocks^37^. The EM region was affected by the NW-SE, NNE-SSW, NE-SW, and NNW-SSE extensional tectonic phases^34^.

### Types of gold mineralization in EM area

Gold mineralization and occurrences encountered in the study area show definite common characteristics and are mainly classified as hydrothermal vein-type gold deposits and occasionally as meta-somatic alteration types. The scenario of the genesis of that mineralization is simply interpreted as follows:

- The source of gold, in general, is the sulphide –rich basic rocks such as the Gabbros and the basic metavolcanics.
- Granitic rocks that intruded later (younger granites) when invaded these basic rocks, the sulphide minerals such as pyrite, chalcopyrite and chalcocite …etc., are then melt due to the thermal heat transfer from the granitic intrusions and the accompanying hydrothermal fluids take the melted gold and re-deposited it again in the hydrothermal qz-veins that are coming from the granitic residual melt and cut through the country rocks of the Gabbros and the metavolcanics at or quite near the contact zone.
- An interaction occurs between the hydrothermal fluids and the surrounding basic country rocks resulting in the formation of a wide alteration zone of meta-somatic alteration in both granite and country rocks. This zone contains gold mineralization as well due to the fixing and adsorption of gold by iron oxides and oxidized sulphides. It is worth mentioning that both processes produce both types at the same time or at least subsequently.

### Surface lineaments analysis

**Fig. 4:**
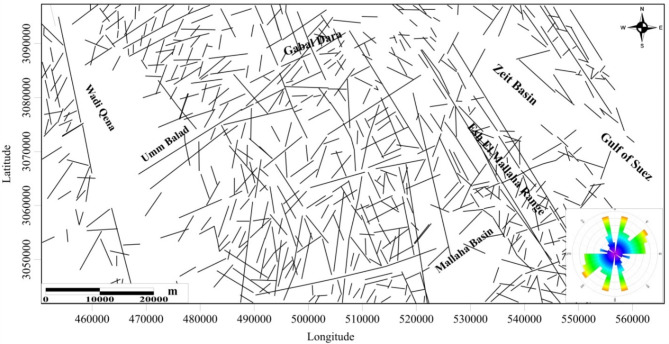
Structural lineaments map of EM area as extracted from the surface geologic map supported by geological field studies, NED, Egypt^32^.

The Structural lineaments map of EM area is extracted from the surface geologic map, NED, Egypt (Fig. 4). The region’s main surface structural trend NW-SE is the dominant trend, followed by NE-SW and NNE-SSW. Less common are the WNW-ESE, NW-SE, and NNW-SSE patterns^34,35^. The progressions of the faults in the EM region varied, signifying several tectonic events. Normal faults are the fault type of the NW-SE fault trend, which is originally started as a strike-slip fault old trend inherited in the basement rocks and rejuvenated as normal faults during the Red Sea- Gulf of Suez rift tectonics.

The NW-SE fault trend was identified on the interface between and cutting the metagabbro-diorite complex and granites^35–37^. The NE-SW fault trends are dextral strike-slip faults, and the N-S fault trend is a dextral strike-slip fault in the EM area, cutting all types of rock. The northern portion of the EM area, the Gabal Dara (GD) area, has iron oxide, Au-Cu mineralization (Fig. 4). It is commonly found in small amounts in quartz veins and the surrounding alteration haloes along the metagabbro-diorite-monzogranite rock contact.

There are exposed veins in the metagabbro-diorites that usually extend NE-SW and NW-SE^34,35^. According to^37^, metagabbro-diorite rocks typically fracture in the NNE-SSW and N-S directions. Auriferous quartz veins and simple dykes enriched with Cu minerals can be found in several of these cracks. Mineralized quartz veins can be found close to granite formations and in metagabbro-diorite rocks. The veins are structurally controlled, with occupied zones, and fracture fissures. A significant source of possible gold in the study’s EM region is the occurrence of the Umm Balad significant gold mines in a particular geological context, which is situated in the centre to the west of the EM area.

The EM area has witnessed an abundance of geological, structural, and mineralogical contributions, especially throughout the western section that comprised the Gabal Dara (GD) and Umm Balad districts^38^. From January 2008 to May 2009, the SMWG Company carried out a geological investigation in the permitted district of Um Balad^38^. They discovered a range of Au concentrations (0.6–13 ppm) in up to 0.5 m thick auriferous quartz veins. They proposed that the discovery of an inadequate quality open-pit mines type deposit would be possible because of the extensive area of the gold mineralization.

### Geophysical dataset

**Fig. 5:**
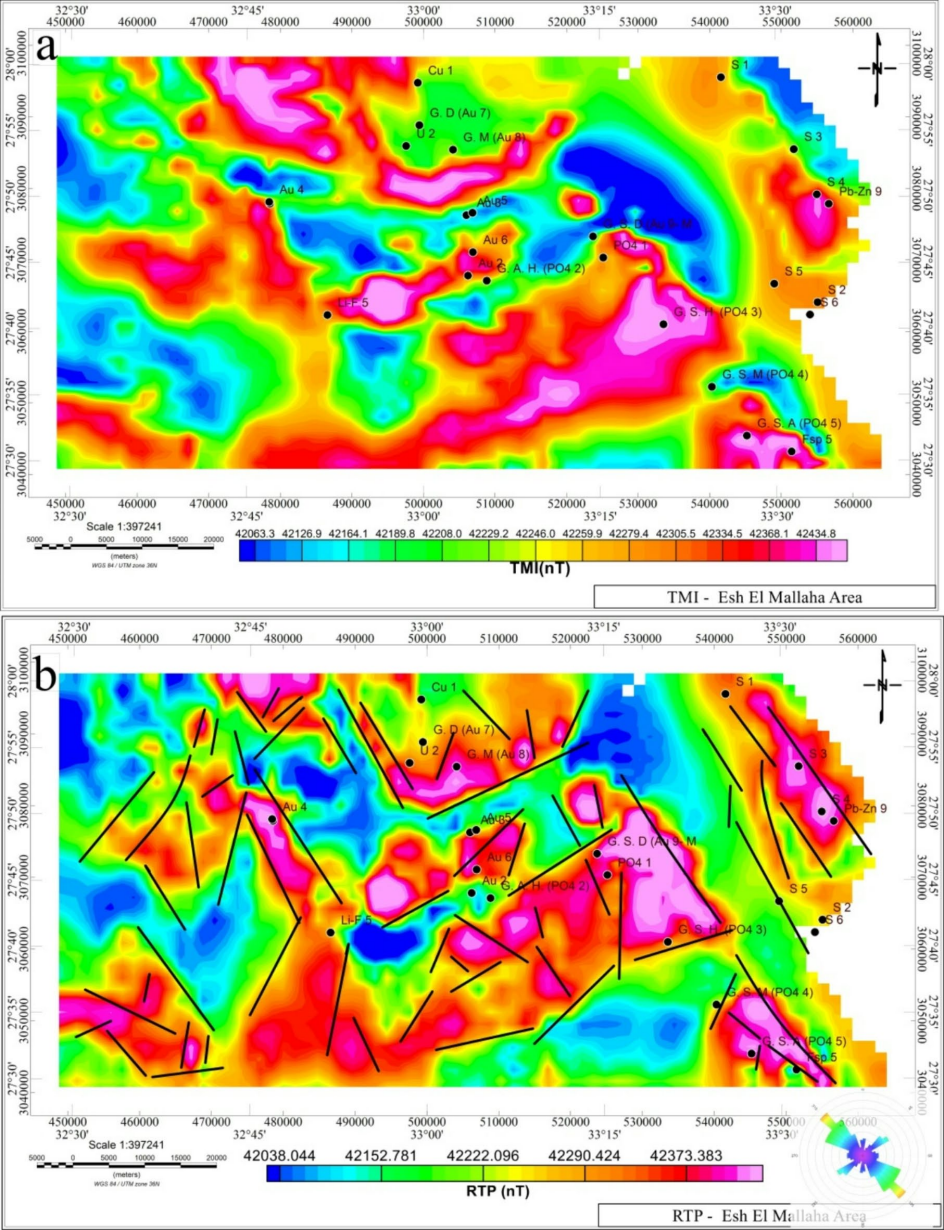
(**a**) Total magnetic intensity anomaly map (TMA) of Esh El Mallaha as redrawn from the original survey conducted by^20–22^, (**b**) the reduced-to-pole (RTP) anomaly map with interpreted lineaments posted based on the magnetic texture. To create the maps, Oasis Montage V 8.4 software was used https://www.seequent.com/products-solutions/geosoft-oasis-montaj/.

The total magnetic intensity (TMI) map (Fig. 5a) is the fundamental data of the current study. It was conducted as a result of an extensive survey conducted by^20–22^. The Varian VIW 2321G4 Single-Cell Cesium Vapor Magnetometer’s survey parameters are as follows: Terrain Clearance altitude 120 m, Traverse = 1.0 Km, Tie = 10.0 Km, Traverse = 45/225, Tie = 135/315, Average TMI 42,425 gammas, Declination 2^o^ E, and Inclination 39.5^o^ N. Following the digitization of the data, the digital map has been processed through several techniques, including the MMTs technique and reduced to pole (RTP). To ensure this filter is properly designed, a uniform grid of specific cells is utilized to create the RTP map. The TMI is transformed into the RTP map as displayed in (Fig. 5b). This indicates that the area, which primarily contains basic granitic rocks, has a rather high magnetic anomaly. The EM region’s centre, northern, eastern, northeast, and southeast portions all indicate a significant high magnetic anomaly. Additionally, in the south of the western portion of the EM region, some anomalies associated with Wadi Qena formation have been found. These include the red beds of the Oligocene sandstone, the hematic sandstone of Nubia formation, and the basaltic flows that cut them as sills and dykes.

## Methods and techniques

### Magnitude magnetic transforms (MMTs)

In brief, the MMTs are generated from TMA^5,16,17^. The anomalies provided by these MMTs are easier to explain and more comparable to the magnetic source’s accurate horizontal location than the measured anomalous magnetic field itself. These characteristics are similar to the conventional magnetic transformations of the analytic signal (AS), the pseudogravity field, and (RTP); but, these MMTs offer a variety of additional advantages. The first-order horizontal derivatives are used to calculate them. Furthermore, unlike the AS, their pattern is independent of the direction of the geomagnetic field vector and is more stable than the RTP’s at low magnetic latitudes.

Three different classifications of MMTs are based on the field derivative order^5,16,17^. The (T_a_) transform, a derivative of the magnetic potential in the same order as the measured magnetic field, is part of the first group. The magnetic anomalies observed with this (Ta) transform are identical to those in the measured field, but they are more precisely localized above the sources.

The second includes the R, E, and Q transforms, which include the first-order derivatives of the observed magnetic field. This group of transforms assists in identifying shallow magnetic sources with identical anomaly shapes and values. They are connected by the formula^17^:

1 $$\:{\text{E}}^{2}\:=\:\frac{{\text{Q}}^{2}+{\text{R}}^{2}}{2}$$

In this study, the (E) transform indicates the second group. The final and the third group consists of the second-order derivatives of the observed magnetic field, including the (L) transform. For the shallowest magnetic causative bodies, it is the most sensitive transform^5,16,17^.

The anomalous magnetic field’s magnitude, ΔT, determines the magnitude transforms^17^. According to^17^, Specify the following definitions for the Laplacian (Eq. 7), gradients (Eqs. 4–6), and magnitude (Eq. 3):

2 $$\Delta {\text{T}}={\text{ }}\left| {{\text{ }}{{\text{T}}_{\text{a}}}+{{\text{T}}_0}} \right| - \left| {{\text{ }}{{\text{T}}_0}} \right|{\text{ }}={\text{ }}\left| {{\text{ }}{{\text{T}}_{\text{m}}} - {{\text{T}}_0}} \right|$$

The observed magnetic field magnitude (T_m_) and normal geomagnetic field vector (T_0_) yield the following MMTs:

3 $${{\text{T}}_{{\text{a }}({\text{x}},{\text{ y}})}}={\text{ }}{\left( {{{\text{X}}_{\text{a}}}^{{\text{2}}}+{\text{ }}{{\text{Y}}_{\text{a}}}^{{\text{2}}}+{\text{ }}{{\text{Z}}_{\text{a}}}^{{\text{2}}}} \right)^{1/2}}$$

4 $${{\text{E}}_{({\text{x}},{\text{ y}})}}={\text{ }}{({\nabla ^{\text{2}}}{{\text{T}}_{\text{a}}}^{{\text{2}}})^{{\text{1}}/{\text{2}}}}/{\text{2 }}={\text{ }}{({\text{ }}|\nabla {{\text{X}}_{\text{a}}}\left| {^{{\text{2}}}+{\text{ }}} \right|\nabla {{\text{Y}}_{\text{a}}}\left| {^{{\text{2}}}+{\text{ }}} \right|\nabla {{\text{Z}}_{\text{a}}}{|^{\text{2}}}/{\text{ 2 }})^{{\text{1}}/{\text{2}}}}$$

5 $${{\text{R}}_{({\text{x}},{\text{ y}})}}={\text{ }}|\nabla {{\text{T}}_{\text{a}}}\left| {{\text{ }}={\text{ }}} \right|{\text{ }}{{\text{X}}_{\text{a}}}\nabla {{\text{X}}_{\text{a}}}+{\text{ }}{{\text{Y}}_{\text{a}}}\nabla {{\text{Y}}_{\text{a}}}+{\text{ }}{{\text{Z}}_{\text{a}}}\nabla {{\text{Z}}_{\text{a}}}|/{{\text{T}}_{\text{a}}}$$

6 $${{\text{Q}}_{({\text{x}},{\text{ y}})}}={\text{ }}{({{\text{T}}_{\text{a}}}{\nabla ^{\text{2}}}{{\text{T}}_{\text{a}}})^{{\text{1}}/{\text{2}}}}={(|\nabla {{\text{X}}_{\text{a}}}\left| {^{{\text{2}}}+{\text{ }}} \right|\nabla {{\text{Y}}_{\text{a}}}\left| {^{{\text{2}}}+{\text{ }}} \right|\nabla {{\text{Z}}_{\text{a}}}{|^{\text{2}}} - |\nabla {{\text{T}}_{\text{a}}}{|^{\text{2}}})^{{\text{1}}/{\text{2}}}},$$

and

7 $${{\text{L}}_{({\text{x}},{\text{ y}})}}={\nabla ^{\text{2}}}{{\text{T}}_{\text{a}}}={\text{ }}(|\nabla {{\text{X}}_{\text{a}}}\left| {^{{\text{2}}}+{\text{ }}} \right|\nabla {{\text{Y}}_{\text{a}}}\left| {^{{\text{2}}}+{\text{ }}} \right|\nabla {{\text{Z}}_{\text{a}}}{|^{\text{2}}} - |\nabla {{\text{T}}_{\text{a}}}{|^{\text{2}}}){\text{ }}{{\text{T}}_{\text{a}}}$$

The magnetic field components X_a_, Y_a_, and Z_a_ indicate north, east, and vertically downward, respectively, while (a) indicates anomalous. Furthermore, in the 2-D situation, the three gradients R, E, and Q are equal and the Laplacian L = R^2^/Ta^5,16,17^.

As previously mentioned earlier, the RTP map is generated only if the direction of the magnetization vector (D, I) and the direction of the geomagnetic field (D_0_, I_0_) are known^39^. When only induced magnetization is present (I = I_0_ & D = D_0_) or when the magnetization direction (D, I) is known and constant throughout the area of interest, the RTP transform provides anomalies centered on the horizontal extends of the appropriate bodies. When (D, I) is unknown and there is a considerable remnant magnetization, interpreters typically assume that I = I_0_ and D = D_0_. This will lead to inaccurate anomalies at the maximum or peak that is not centered over the magnetization source. Fortunately, the magnetization direction (D, I) exhibits no influence on the estimation of the positive transform (Ta)^40^.

### Fractal/ multifractal and singularity index

In the geosciences, fractal geometry has been employed extensively since the 1980s^41^. In addition to investigating complex geological structures, engineering, economic geology, mineral exploration, geophysical analysis, and geochemical anomaly separation for identifying mineral concentrations^42^, fractal/multifractal modelling is used in the geosciences to classify a wide range of properties^43–48^. According to^45,49^, these methods have several benefits; among them is the ability to determine the geometric structure of areas using parameter distribution. Both the Concentration-Area (C-A) methodology and the local singularity analysis (S-A) technique, which were established by^49,50^, respectively, are very useful methods in earth sciences that can be applied to mineral prospecting.

Traditional statistical techniques merely distinguish between anomalous classes; they ignore the geographical distribution and autocorrelation structure of the data. When interpolating and finding trends, geostatistical interpolation models, on the other hand, can analyse spatial structures while taking into account their spatial properties. These interpolation methods’ main drawback, meanwhile, is that they flatten or smooth out the anomalies, rendering areas of weakness undetected or leading them to overlap with high background values. Many significant exploration targets may be missed as a result of this critical problem, which is becoming increasingly prevalent in mineral prospectivity mapping under complex geological conditions^50,51^. Fractal and multifractal approaches, on the other hand, consider both the geometric shape of the anomalies and the spatial distribution of the data. Furthermore, they employ all available data without altering them, making them far more effective in precisely distinguishing between anomaly classes and background^52,53^. Among these techniques, the integrated Fuzzy AHP-VIKOR method proposed by^54^, where the authors generated a potential map of gold in the study area by implementing three Mineral prospectivity mapping techniques, including conventional VIKOR, Modified VIKOR, and multi-class index overlay.

In this work, we use a variety of fractal and multifractal models and singularity analysis S-A applied to the MMTs transforms instead of the traditional RTP transforms to identify geophysical anomalies in magnetic data acquired in the Esh El Mallaha EM district of Egypt’s Northern Eastern Desert. This enables us to identify potential mineralization prospects and new exploration areas. In mathematical terms, the three statistical approaches that were applied can be summarized as follows:

### Inverse distance weighted (IDW)

IDW is among the most widely used scatter point interpolation methods in ArcGIS software. Closer points should have a greater impact on the interpolating surface than further points, according to the basic concept of IDW. The average weighted of the data available at known places is used to generate the values at unknown locations^55^. IDW is a method of moving averages that prioritises nearby data from observations over far-off values for interpolation, according to^56^. IDW possesses the effectiveness of a mining operation depending on the precision of reserve evaluations and mineral categorisation distributions^57^.

### The concentration-area (C-A)

The C-A fractal model was proposed by^49^ and has been used to identify geochemical, geophysical, and mineral exploration anomalies in background data^45,58^. Analysed multispectral images of porphyry systems using this method. The spatial relationship values of each evidence deposit were later separated and identified using fractal modelling (C-A) by^49^, who also used the break-points between straight path sections C-A log-log plots to establish boundaries for enrichment anomalies. The C-A fractal model may distinguish between background values and anomalies in evidence layers when describing a distribution component in a specific zone. A(ρ) is the region with concentration values above the contour value ρ, a1 and a2 are standard exponents, and v is the threshold, Eq. (8).Cheng et al.,^49^ explains the framework of the model. To determine the area A(ρ) for a given ρ, multiply the cell area by the number of cells that have pixel values greater than ρ. To divide pixel data into related components, log-log graphs utilize slope variances with ρ quantities as cut-offs^49^. In the present work, we use the C-A fractal model to identify potential mappings that require further exploration.

8 $${\text{A}}\left( {\rho {\text{ }} \le {\text{ v}}} \right) \propto \rho ^{{( - {\text{a1}})}} ;{\text{ A}}\left( {\rho {\text{ }} \ge {\text{ v}}} \right) \propto \rho ^{{( - {\text{a2}})}}$$

### Local singularity analysis ( S-A)

Because of the multi-phase nature of mineralization and the consequences of geological complexity, geophysical and geological data are frequently insufficient with a single statistical distribution form; consequently, traditional multivariate statistical methods are ineffective for exploring and identifying the distribution of significant details in multivariate data^49,59^. The development of technology for fractal and multifractal anomaly detection and extraction, particularly the S-A method for identifying complicated geological anomalies that were hidden in the background but only partially successful. Geologically important mineralization, which is described as the distribution or build-up of massive energy in a restricted region over a relatively short amount of time, results in the formation of anomalies called singularities^49,60^. Local quantitative self-similarity features are often seen in observations with singular distributions. The singularity approach is a potent multifractal modelling technique^61^. To extract and analyse weak and overlapping anomalies that arise throughout the mining process, the local singularity S-A approach is helpful. S-A can be determined in real-world applications by employing the sliding windows method with raster or gridded data as the source. The S-A was constructed using the sliding window method, which was initially suggested by^49^ and has significant geological consequences. The singularity can be estimated using the following equation^49^:

9 $${\text{X }} = ~~{\text{C}}.\varepsilon ^{{({\text{a}} - {\text{E}})}}$$

where (X) is the elemental contents, (C) is a constant value, (ε) is the cell size, (a) is the singularity value in every location and E is the Euclidian dimension^13,46,47^. The singular value takes into consideration multifractal theory estimations of the local singularity of anomalies. An element is regarded to have a normal background; whenever its oddness singularity index (a) is close to two; when it is less than two, it can be recognized as strengthened and enriched; and when it is more than two, it diminishes^49^.

### MMTs vs reduction-to-the-pole RTP

**Fig. 6:**
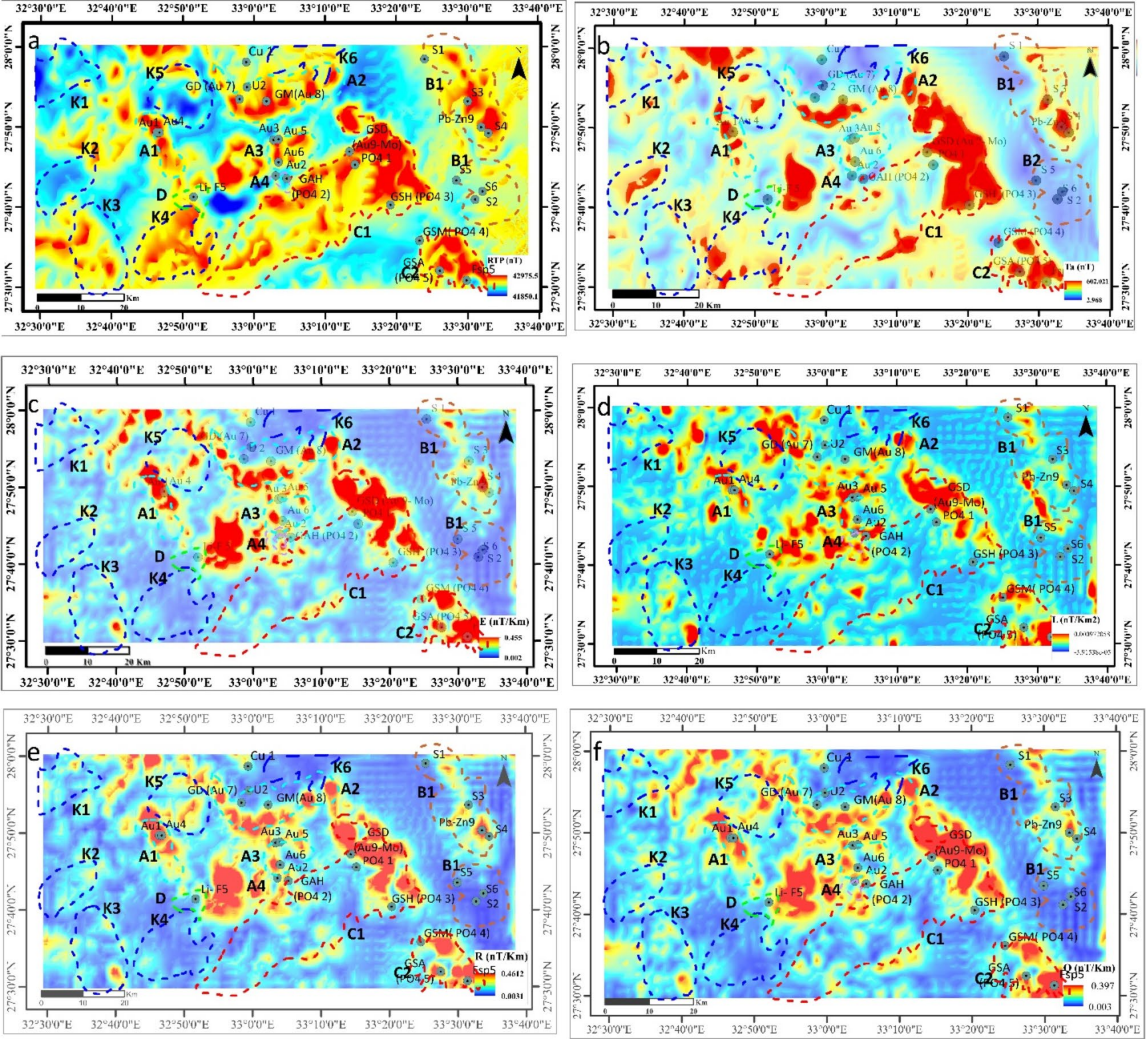
The RTP and T, E, L, R, Q Magnitude Transformers(**a** ) RTP, (**b**) T_a_, (**c**) E, (**d**) L, (**e**) R, and (**f**) Q Maps of EM area, Northern Eastern Desert, Egypt. Dashed zones represent anomalous zones of interest. K’s represents selected anomalies, and Au1, Au2… (Gold mine), Cu1 (Cupper mine), Li (lithium occurrence), Pb-Zn (lead zinc mine), …, etc. To create the maps, ArcGIS Desktop version 10.8 was used. https://www.esri.com/en-us/arcgis/geospatial-platform/overview.

The Ta-transform, or simply T_a_, is the best transform to compare with the RTP in (Fig. 6a & b), according to^5,39,40^. In this case, the RTP and the measured magnetic field ∆T are both of the same order of the magnetic potential. The significant benefits of the transform T_a_ are listed below in (Table 1):

**Table 1:** A comparison between the RTP and the T_a_ -transform.

RTP transform	T_a_ -transform
Prior knowledge of the source magnetization vector’s (inclination I and declination D) and geomagnetic field’s (inclination I_0_ and declination D_0_) directions is required for the RTP estimation to operate effectively. For the T_a_ calculation, only the direction (I_0_ and D_0_) of the geomagnetic field is required.	Whereas the RTP maps, like the measured field, can have both positive and negative values, T_a_ only has nonnegative values. This makes it easier to compare the T_a_ maps with those from geological sources and to interpret them visually.
I = I_0_ and D = D_0_ are the main limiting assumptions for applying the RTP transformation, which states that the source magnetization is exclusively induced. This assumption will result in inaccurate RTP field mapping, which will affect how the RTP anomalies are interpreted both quantitatively and qualitatively. This is particularly risky because many inversion techniques employ the RTP field instead of directly inverting the observed magnetic field, as in^5^. As a result, inaccurate estimations will be generated by any ineffective inversion technique.	No assumptions regarding the nature of source magnetization are generated by the transform T_a_.
	T_a_ has greater potential than RTP for simplified magnetic field representations especially when the same map has sources that have distinct magnetization directions or remaining source magnetization [5][39][40]
Low magnetic latitudes produce instability in the RTP operator. At latitudes I_0_ = 20° and D_0_ = 0, the RTP transform maps are deformed and difficult to comprehend. Special stabilization procedures for low magnetic latitudes^62,63^ are needed to calculate interpretable RTP maps, which result in neater-looking but occasionally inaccurate maps. The issue of the frequently inaccurate assumption of induced magnetization additionally pertains up.	The T_a_ calculation can provide accurate and well-centered maps.
Low magnetic latitudes cause instability in the RTP transfer function^16^.	The frequency domain T_a_ estimate techniques have a denominator that is one degree below the RTP transfer function’s denominator^16^.
Special methodologies are required to calculate the RTP throughout extensive regions^64^.	The same is true for the T_a_ transforms [65], however the RTP requires more complicated processes than the T_a_ computation. Another challenge with RTP computation across large regions is the assumption that source magnetizations are solely induced, which is rarely achieved.
	In regard to signal dependence, T_a_ could more accurately represent the magnetic field that revolves on each of the associated sources^1^.
In contrast to the measured magnetic field, the centricity is substantially better for the 3D situation, even though the magnetic anomalies are not precisely centered over the magnetic sources^17^.	When induced-only source magnetization is present, the transform T_a_ to RTP’s primary drawback occurs. The primary benefits of MMTs are their centricity to the sources of their anomalies and their shape regarding the geomagnetic field’s orientation (D_0_ & I_0_).
A complicated and memory-intensive process is the RTP computation.	Simple calculation

## Results and discussion

The observed magnetic anomalies in the area are primarily linked to gold mineralization. The presence of sulphides exhibiting ferromagnetic and paramagnetic properties within the quartz veins is a significant contributor to the high magnetic anomalies associated with these veins. Additionally, hydrothermal fluids from the later stages of granitic intrusions introduce substantial amounts of iron oxides, which, upon interaction with the surrounding country rocks, create extensive metasomatic alteration zones that are reflected in the magnetic records. It is noteworthy that the quartz veins themselves do not exhibit any magnetic response. However, their association with sulphides and iron-rich alteration zones results in their inclusion within the anomalous magnetic zone. This relationship underscores the importance of both the mineral composition and the geological processes in understanding the magnetic anomalies in the region.

The EM area’s MMTs are shown in (Fig. 6). The transforms demonstrate an obvious connection between the magnetic anomaly associated with the potential magnetic sources and their horizontal projection. Additionally, over these magnetic sources, the anomalies are concentrated, and MMTs in these processes show a pattern that is exactly the same as the structures of field source anomalies.

The RTP field shows several elongated anomalies trending (NW-SE) (Fig. 5b, & 6a). The main selected high RTP anomalies (in red colour) reach 42,975.5 nT, especially in gold (Au) mine zone locations. The area can be divided into several magnetic anomaly zones (areas bounded by dashed lines) namely **A1**,** A2**,** A3**, and **A4**. **A1** zone contains Au_1_ and Au_4_ gold mines that are geologically represented by older gray granite, **A2** zone contains Au_7_, and Au_8_. This zone comprises also uranium mine U_2_ with gold mines that are composed of acid metavolcanics, and younger granite. **A3** zone has Au_3,_ Au_5,_ and Au_6_ gold mines formed of older gray granite, and Dokhan volcanic (Figs. 3 and 6a).

While zone **A4** has Au_2_ that represents a low RTP anomaly region (about 42,150 nT) due to its complex geological structure and weathered rock units (Hammamat classics, metgabbro-diorite complex rocks surrounded by older gray granites). When applying the MMTs on the TMA anomaly, some obvious changes occurred to the anomalies. When comparing T_a_ transform with RTP (Fig. 6a & b), the zones **K1**,** K2**,** K5** are appeared for the first time in the T_a_ map (Fig. 6b), and (Fig. 6a) Conversely, K3, K4, K6 are disappeared in anomaly T_a_ map (Fig. 6a & b). Moreover, the size and magnitudes of other anomalies can be observed (see zones B1, B2, C1, A2). This may be related to that the magnitude of the T_a_ transform is considerably more stable than the RTP operator; consequently, the modified anomalies are not distorted in the direction of geomagnetic declination^16^. E, R, and Q transform are similar in their anomaly patterns but the anomaly amplitudes are sharper than the L transform (Fig. 6c-f).

Zone **B1** is characterized by a high RTP anomaly that overlays rocks of sulfur content at (S1, S3, and S4). This also includes (Ph-Zn9), which is close to (S4) and geologically represented by the Shagra Formation, with the complexity of younger granite parts. **B2** is characterized by low RTP anomaly that also overlays rocks of sulfur content (S2, S5, and S6) and geologically represented by Shagra Fm., and wadi alluvium surrounded by sabkha deposits (Figs. 3 and 6a). The T_a_ transform has a sharp response on the edges, while the E, R, Q transforms attain their clear peaks over the anomalies centre. The L-transform is characterized by fewer numbers of anomalies than the other transform. In the center of the EM area, zone C1 is characterized by an intermediate to high RTP anomaly, which contains phosphate deposits at (PO_4_ 1, PO_4_ 2, and PO_4_ 3). The Wadi alluvium and Umm Mahara Formation, which are surrounded by the Thebes group, represent their geological representatives. A feldspar mine with a Fsp 5 is located within the (C2) zone, which is defined by a high RTP anomaly in the northeastern portion of the EM area, which also includes phosphate deposits at (PO_4_ 4, PO_4_ 5).

It is geologically represented by Umm Mahara Formation, Wadi alluvium, and Taref Formation overlayed by Qusier Formation and surrounded by the Thebes group. At zone (D), low to middle RTP anomaly are dominant. It comprises (Li-F 5) mine and geologically represented by Wadi alluvium, and Dokhan volcanic. The (E) zone at Gabal Dara area (GD) in the north part of EM area this zone is characterized by intermediate RTP magnetic anomaly overlying the Copper (Cu 1) mines, which is geologically represented mainly by younger granite (Figs. 3 and 6a). Zones (C1) and (C2) are shrunk and centred over the source anomalies when applying MMTs. (Figs. 6b-f) exhibit each of the five MMTs’ maximum values (T, E, L, R, and Q).

Since the T_a_ transform only includes first-order terms, as established by Eq. (3), it has non-resolved anomalies at K1, K2, K3, K4, and K5, but the E, L, R, and Q transforms, as indicated in (Fig. 6b-f), have significantly higher frequencies (higher derivatives) and a greater number of shallower anomalies.

R and Q transform arithmetic means are simply the E transform, as illustrated in Eq. (4) (Fig. 6c, e, & f)^1^. The maps provide greater insight into the shallow sources than the T_a_ transform, with more anomaly visibility or recognition, because E, R, and Q are the three transforms that are the 1st derivatives of ∇Xa, ∇Ya, and ∇Za. The Q transform, on the other hand, improves coherence for some trends, although it is unclear whether the difference is due to anomaly type or distribution^1^. The L transform, which is described as a function of 2nd order derivatives reflects the shallower anomalies in the study area. This provides significant variations from the E, R, and Q results. The T_a_ transform often gets better edges of these anomalies, as illustrated in the anomalies located at K1, K2, K4, K5, A2, B1, and C1 in (Fig. 6b).

### Multifractal and singularity analysis of MMTs

To enhance interpretation and improve spatial targeting of the mineralized zones, the Concentration-Area C-A^49^, Singularity-Analysis S-A^50^, and MIDW^56^ are calculated utilizing the MMTs. (Figures 8 and 9) demonstrate Multifractal C-A and singularity analysis S-A of MMTs in the EM region of Egypt’s NED based on MIDW interpolation observations generated using the ArcGIS 10.8 program (https://www.esri.com/en-us/arcgis/products/arcgis-desktop/overview).

**Fig. 7:**
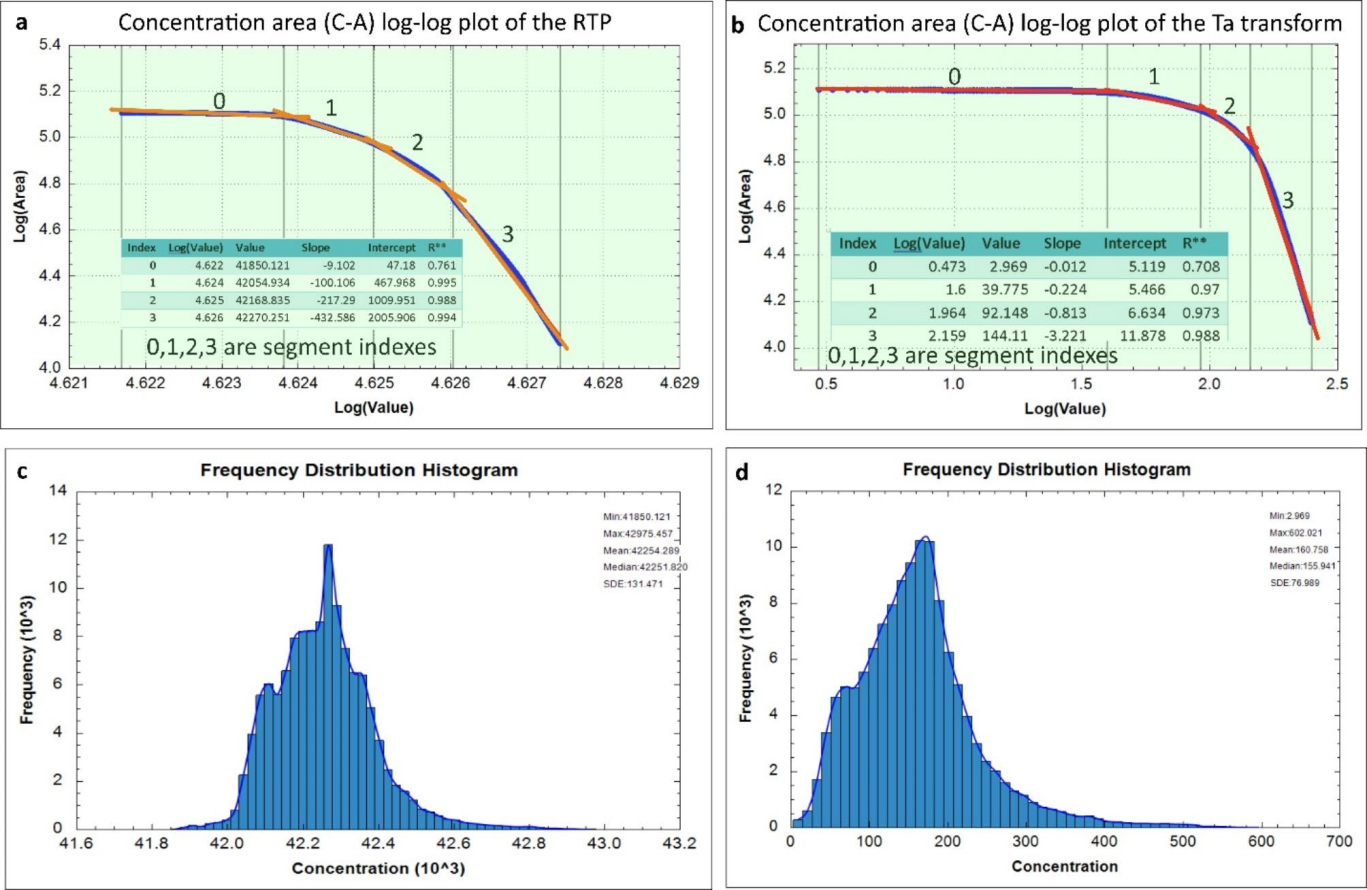
(**a**,**b**) C-A log-log plot with data listing, (**c**,**d**) data histogram. To create the maps, ArcGIS Desktop version 10.8 was used. https://www.esri.com/en-us/arcgis/geospatial-platform/overview.

The Fractal C-A /T_a_ is useful in the identification of anomalies. C-A is a highly useful tool for characterizing the distribution of concentrations of elements and separating anomalies from the background. (Fig. 7) shows the C-A log-log plot for the T_a_ transform, which was made using the estimated T_a_ transform model based on TMI (TMA) data. regarding the basis of breakpoints and linear segments.

**Fig. 8:**
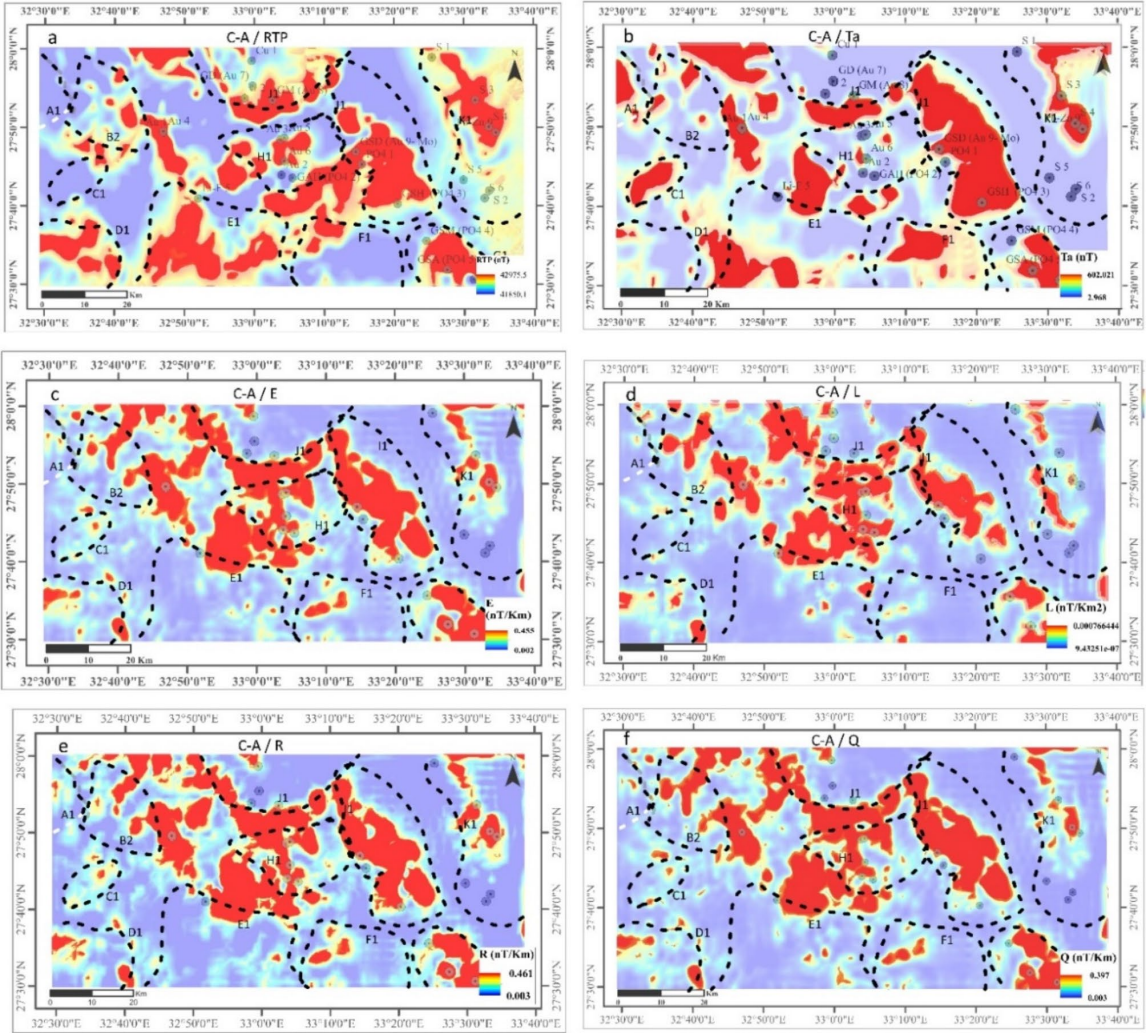
Comparison of the C-A of RTP and that of the MMTs Transformers. (**a**) RTP, (**b**) T_a_, (**c**) E, (**d**) L, (**e**) R, and (**f**) Q Maps of EM area, Northern Eastern Desert, Egypt. To create the maps, ArcGIS Desktop version 10.8 was used. https://www.esri.com/en-us/arcgis/geospatial-platform/overview.

The C-A / RTP in (Fig. 8a), signifies and highlights magnitudes than in RTP (Fig. 6a) itself, so the anomalies are further identified with intensities reaching 42,795.5 nT, the zones that have very strong C-A /RTP anomalies are (B1, E1, D1, G1, H1, I1, J1, and K1) most of them contain or close to known historic mines. This economic perspective renders these anomalies extremely promising. The application of the C-A /MMTs transform maps enhances the identification of anomalies, resulting in a clearer distribution that converges more closely to the locations of the mines (Fig. 8a-f). There are notable variations in the anomalies observed in the CA of RTP (e.g. at J1).

These anomalies exhibit a more pronounced presence in the C-A of RTP compared to their representation in the C-A of the MMTs, as previously indicated (Fig. 6b-f). This discrepancy may be attributed to a potential remanence effect that influences the RTP field while diminishing its impact in the C-A of the MMT field. Consequently, the application of C-A for the first time on MMTs to demonstrate the distribution of clustered anomalies, particularly those with centricity above the distributed mines within the EM area, represents an innovative approach to mineral exploration (Fig. 6b-f).

The anomaly distributions of C-A of E, R, and Q exhibit similarities and demonstrate a strong correlation with the mine locations at H1, J1, K1, and G1 (see Fig. 8c, e, & f). Likewise, the L transform shows a comparable extracted anomaly distribution (see Fig. 8f).

The C-A of MMTs typically provides a clear identification of significantly spatially distributed anomalies, particularly those with magnitudes greater than 602.021 nT for the T_a_ transform. NE-SW, NNE-SSW, ENE-WSW, and ENE-WNW are the main anomalous trends observed. Mine distribution is closely associated with the majority of the distinctive C-A anomalies. Specifically, the most prominent anomaly correlates with the presence of three mines in the northeastern region, two in the southeastern area, three in the eastern section, one in the northern part, and six mines located in the central to western region.

### Singularity analysis

**Fig. 9:**
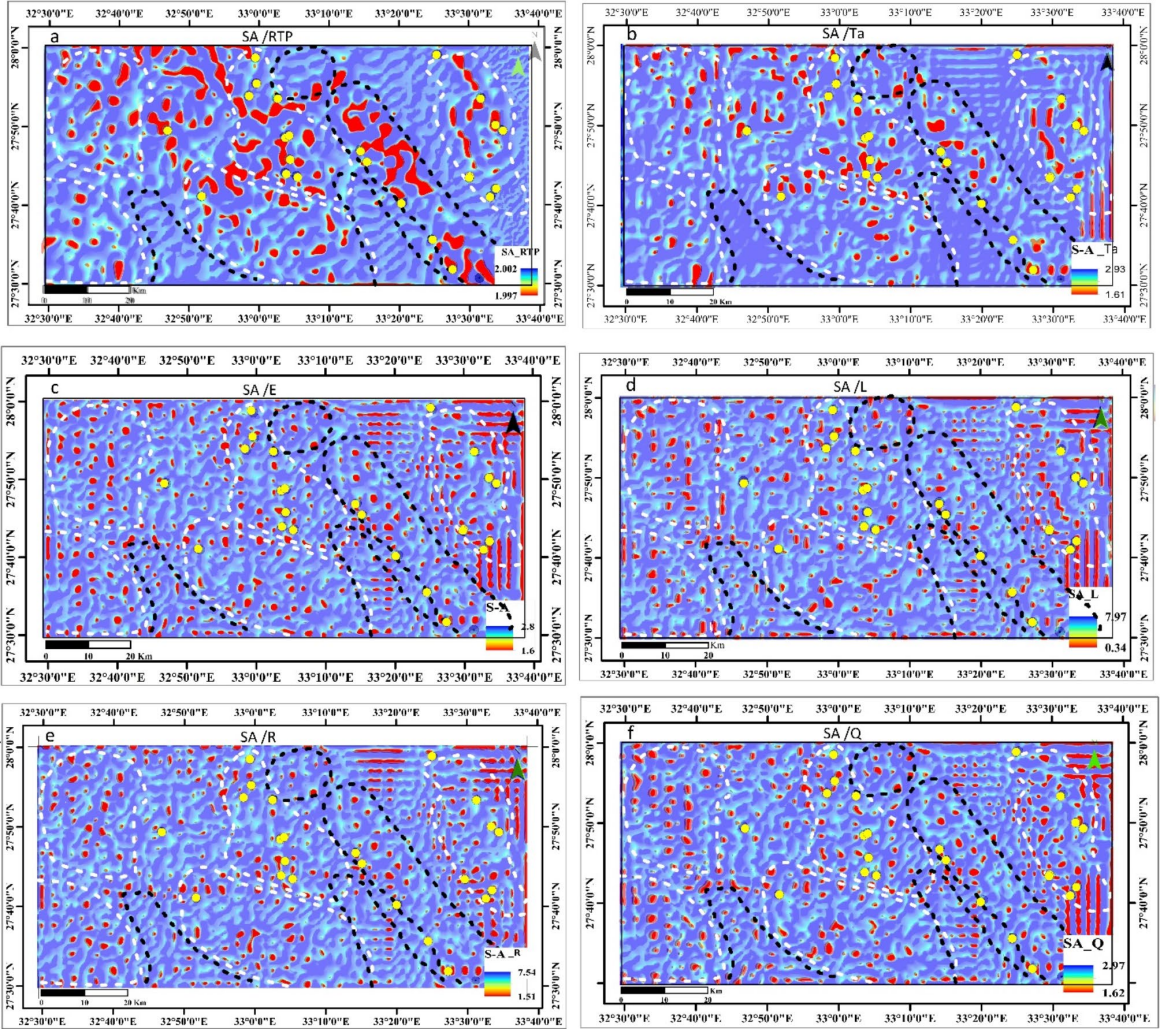
Comparison of SA RTP with Transformers. (**a**) RTP, (**b**) Ta, (**c**) E, (**d**) L, (**e**) R, and (**f**) Q Maps of EM area, Northern Eastern Desert, Egypt. Dashed white zones are zones of mineralization. While black zones are expected potential zones. The yellow circles are the known gold mines. To create the maps, ArcGIS Desktop version 10.8 was used. https://www.esri.com/en-us/arcgis/geospatial-platform/overview.

The efficacy of the singularity S-A method is clearly illustrated in the singularity maps (Fig. 9). Minor and localized anomalies have been detected throughout the EM region, with the boundaries between larger anomalies becoming more pronounced and defined. A notable magnetization anomaly was anticipated due to the presence of Precambrian basement formations, including granitic, Dokhan volcanic, meta gabbro-diorite complex rocks, as well as acid metavolcanic and metavolcanic units. Additionally, there are other geological units associated with mineral deposits in the EM area; however, the RTP maps did not reveal any anomalies in certain sections of this region, suggesting the presence of a concealed anomaly. The singularity approach was used to discover local irregularities in the mentioned region that had previously been hidden owing to the complexity of geology. The S-A is typically capable of distinguishing this local anomaly from the regional one, which demonstrates a favourable distribution of several new prospects at various locations associated with the established mines. Recent advancements in the detection of subtle anomalies against the background involve the singularity mapping technique proposed by^49^. Finding weak anomalies in complicated geological settings or areas obscured by overburden is considerably simpler with this multifractal technique.

A multifractal model is frequently used by the Singularity analysis (S-A) technique to examine the local arrangement of mineralization. It can also detect the occurrence and enrichment of mineralization features. Therefore, in areas with complex geological conditions, the singularity mapping technique can be beneficial for identifying unexpected mineral reserves with a (S-A) of less than 2, where small anomalies may be hidden by a strong background. The T_a_ transform was subjected to this technique (Fig. 3b). Based on the size of the unit cell, the scale of the S-A of interest, and the size of the EM region, the edge sizes and intervals were selected to be used in singularity S-A mapping. This approach aims to identify the local characteristics of geophysical data. As evidenced in the S-A map of the T_a_ transform (Fig. 9), weak and limited anomalies have developed that are nearly spatially correlated with known mineral deposit occurrences in the EM region. In such areas, several magnetic anomalies have been found in the northwest, southwest, western, southern, and northern parts (shown by the yellow & black circles). The sites of mineral deposits that were missed by the C-A fractal techniques are represented by these anomalies.

In summary, compared to other approaches used in this study, the results of the singularity S-A mapping techniques show a high degree of accuracy and are consistent with a variety of geological data found in the studied area. Additionally, the large anomaly’s singularity index S-A is roughly 1.6, offering an extensive amount of opportunity for mineral exploitation.

## Conclusion

Magnetic data have been analyzed using magnitude magnetic transform techniques (MMTs). The newly proposed approach enhances our understanding and interpretation of the data, eliminating the need to know the inclination and declination of the source body previously. The application of multifractal analysis and singularity index on the anomalies transformed by MMTs has led to improved peak resolution and a reduction in the spatial distribution of anomalies. Most of the identified anomalies associated with potential source bodies, such as known mines, are now well-centered and resolved. In particular, the application workflow on EM data shows, in addition, newly detected zones of anomalies of interest, The T_a_ transform reduced the number of peaks, increased smoothness, and simplified the anomalies’ shape.

Compared to the measured observed fields, the MMTs approach significantly improves the visual resolution of complex anomaly patterns. Other peak values show the positions of extra magnetic sources on the R, E, and Q transform maps, which depict certain gradients of magnetic field intensity. The L transform, which is the magnetic field’s Laplacian, reveals a higher concentration of anomalies. The peak shapes that resulted from this alteration are significantly sharper and more closely matched to the origins. As a result, the resolution of clustered peaks is now easier to see and recognize. As a result, the L transform, as a sum of second-order derivatives, mostly reflects the influences of shallower sources.

Together with a combined analysis of geological data and other relevant information, the overall application of the multifractal and singularity analysis on the anomalies transformed by MMTs improves the estimation of Au mineralization zones and the accuracy of magnetic source identification.

## Data Availability

The datasets used and/or analyzed during the current study are available from the corresponding author upon reasonable request.
